# Serum IL-5 levels predict HBsAg seroclearance in patients treated with Nucleos(t)ide analogues combined with pegylated interferon

**DOI:** 10.3389/fimmu.2022.1104329

**Published:** 2023-01-05

**Authors:** Peipei Wang, Zhishuo Mo, Ying Zhang, Chunxia Guo, Trevor Kudzai Chikede, Dabiao Chen, Ziying Lei, Zhiliang Gao, Qian Zhang, Qiaoxia Tong

**Affiliations:** ^1^ Department of Infectious Diseases, The Third Affiliated Hospital of Sun Yat-sen University, Guangzhou, Guangdong, China; ^2^ Guangdong Key Laboratory of Liver Disease Research, The Third Affiliated Hospital of Sun Yat-sen University, Guangzhou, China; ^3^ Key Laboratory of Tropical Disease Control (Sun Yat-sen University), Ministry of Education, Guangzhou, Guangdong, China; ^4^ Department of Infectious Diseases, Union Hospital, Tongji Medical College, Huazhong University of Science and Technology, Wuhan, China

**Keywords:** IL-5, HBsAg seroclearance, cytokines, pegylated interferon, nucleos(t)ide analogues

## Abstract

**Background:**

Knowing about cytokine profile contributes to clarify the underling immune mechanism of HBsAg seroclearance rate increase. This study aims to investigate cytokine changes during nucleos(t)ide analogues (NAs) and peginterferon-α (Peg-IFNα) therapy and their impact on the HBsAg serologic response.

**Methods:**

A total of 78 HBV DNA-negative chronic Hepatitis B (CHB) patients were studied after a lead-in phase of NAs with complete serum cytokines. Serum cytokines (IL-1β, IL-2, IL-4, IL-5, IL-6, IL-8, IL-10, IL-12, IL-17 and TNF-α) were quantified by flow cytometry (FCM) every 24 weeks, before, during and at the end of NAs and Peg-IFNα treatment. Clinical and laboratory data were also taken at the same time. Analysis was performed between cured and uncured groups characterized by HBsAg seroclearance. PBMCs samples from five patients (two in cured group and three in uncured group) were analyzed by FCM.

**Results:**

HBsAg seroclearance was achieved in 30 (38,5%) patients defined as the cured group. In comparison to uncured individuals, cured patients showed similar expressions of serum IL-1β, IL-2, IL-4, IL-6, IL-8, IL-10, IL-12, IL-17 and TNF-α during the treatment of NAs and Peg-IFNα. Compared with the uncured groups, IL-5 was remarkably increased in cured patients. IL-5 at weeks 24 and 48 were associated with HBsAg seroconversion (p=0.033 and 0.027, respectively). PBMCs sample analysis confirmed the predicted value of IL-5 in response to NAs and Peg-IFNα treatment.

**Conclusions:**

IL-5 at weeks 24 and 48 might be used as a biomarker for HBsAg seroclearance in NAs-experienced CHB patients treated with NAs combined with Peg-IFNα. More importantly, exploiting the expression of this cytokine may help to develop a better understanding of the immune pathogenesis of chronic HBV infection.

## Introduction

Seroclearance of hepatitis B surface antigen (HBsAg) (“functional cure”) is regarded as the optimal endpoint of antiviral therapy for chronic hepatitis B virus (HBV) infection (1–3), which can stimulate host immune response to induce variable extent of liver injury. Recent studies have emphasized a higher rate of HBsAg loss (4–6) and lower incidence of HCC (7) among individuals treated with nucleos(t)ide analogues (NAs) and peginterferon-α (Peg-IFNα) combination therapy. HBeAg-negative individuals, with lower baseline HBV DNA and quantitative HBsAg levels, had higher rates of HBsAg seroclearance, suggesting that more patients in the inactive phase achieve functional cure (4, 8). Although the use of these two types of available approved treatments has resulted in a remarkable improvement in the rate of HBsAg seroclearance, they are still facing new issues such as immune tolerance/exhaustion and disease progression. It is vital to identify the factors to further improve clinical management.

The progression of chronic hepatitis B virus (CHB) infection is predominantly mediated by persistent intrahepatic immunopathology. Cytokines play an important role in initiating, maintaining, and regulating immunological homeostatic and inflammation of CHB infection. Interleukin (IL)-2 is associated with the evaluation of functions in T cells, especially in the HBV- specific T cells during the natural course of HBV infection, as well as its dynamics in response to the anti-HBV treatment (9, 10). Elevated INF-γ is accompanied by a decrease of IL-4 from the immune tolerance phase to the inactive carrier phase (11), indicating a shift from Th2 to Th1 responses. A sustained high level or dynamic elevated level of serum IL-6 indicates higher mortality in patients with HBV-acute-on-chronic liver failure (ACLF) (12). Furthermore, higher serum levels of IL-12 were accompanied by HBeAg or HBsAg seroconversion in CHB patients (13, 14).

In addition to the cytokines reviewed above, there are still many other cytokines that have directive implications in the possibility of designing immunotherapies for CHB patients. IL-5, mainly produced by T helper-2 (Th2) lymphocytes, can increase antibody secretion by promoting the differentiation and growth of B cells and enhancing the humoral immune response mediated by Th2 cells. It was also associated with viral response and HBeAg seroconversion after entecavir (ETV) therapy in CHB patients, suggesting serum levels of IL-5 may be an available marker to predict responses to anti-HBV therapy (15). Here, we investigated the expression pattern of cytokines, especially IL-5, in patients with chronic HBV infection, and thereby assessed the relationship between cytokine expression and the effectiveness of NAs and Peg-IFNα combination therapy.

## Methods

### Patients

The study population recruited a total of 95 HBeAg-negative chronic hepatitis B patients from the third Affiliated Hospital of Sun Yat-Sen University, Guangzhou, China from March 2019 to May 2021. All procedures were conducted according to the principles of the Declaration of Helsinki, the protocol and consent forms were approved by the Research Ethics Committee of the Third Affiliated Hospital of Sun Yat-sen University. All participants provided written informed consent. Inclusion criteria included age between 18 and 65 years, NAs treated experience more than 1 year, treatment-naive for interferon, and low hepatitis B surface antigen levels: HBsAg < 1500 IU/ml. The exclusion criterion for this study included advanced liver cirrhosis, presence of hepatocellular carcinoma or other malignancies, other causes of liver disease or mixed causes, human immunodeficiency virus co-infection, and known serious psycho-pathological disorders.

All patients received peg-interferon α-2b (Pegberon, Amoytop Biotech Co., Ltd., China.) treatment based on the chronic administration of NAs for 48 weeks. The course could be shortened if a patient showed HBsAg seroclearance (HBsAg<0.05IU/ML) and lasted more than 12 weeks during the follow-up. The patients who achieved HBsAg seroclearance during the course of treatment was defined as cured group. Conversely, if the patient had detectable serum HBsAg at the end of treatment (EOT), the given patient was attributed to the uncured group. Synchronous serum samples were collected before and during Peg-IFNα treatment at 12-week intervals for 48 weeks. Blood sample (10 ml) from each patient was collected in the EDTA-treated tube. Plasma was collected after centrifugation, then peripheral blood mononuclear cells (PBMCs) were isolated by Ficoll-Hypaque density gradient centrifugation. All samples were stored at −80°C and used for the present study.

### Serological parameters

Blood routine indexes were assessed using an automated blood cell analyzer (Sysmex XN-3000, Kobe, Japan). Biochemical indexes of liver function were detected by an automatic biochemical analyzer (HITACHI 7600, Japan). HBeAg, HBeAb, HBcAb, and HBsAb level testing were performed using Roche Cobas E601 automatic electrochemiluminescence assay (Roche Diagnostics, IN, Germany). HBsAg was measured with Elecsys HBsAg II Quant reagent kits (Roche Diagnostics, Indianapolis, IN, USA). Serum HBV DNA was assayed with a Roche COBAS AmpliPrep/COBAS TaqMan HBV Test v2.0 (Roche Molecular Diagnostics, Branchburg, NJ, USA). These analyses were performed at the laboratory department of the third Affiliated Hospital of Sun Yat-Sen University and all procedures were performed following the manufacturer’s instructions.

### Cytokine assays

Serum samples of patients were collected by centrifugation of blood at 3200 rpm for 10 min and stored immediately at -80°C until use. Using FACS Calibur flow cytometer (BD, San Diego, USA), we detected 10 cytokines (IL-1β, IL-2, IL-4, IL-5, IL-6, IL-8, IL-10, IL-12, IL-17 and TNF-α) with the cytokine detection kit (multiple microsphere flow immunofluorescence method; Changsha Weimi Bio-Tech Co., Ltd., China) by following manufacturer’s instructions.

### Flow cytometry

Thawed PBMCs samples were taken from five patients (two in cured group and three in uncured group). A total of 15 blood samples were collected at 3 time points: day 0 (the day before), week 24, and week 48 of treatment. Samples were washed 3 times by 1x PBS with centrifugation at 400 g for 6 min and counted for cell numbers. 500,000 cells per tube were then stained for 30 min on ice with antibodies. For flow cytometric analyses, the following antibodies were used: CD3 (CD3-FITC; BioLegend, cat#300305), CD4 (CD4-APC-Cy7; BioLegend, cat#317418), CD8 (CD8-PerCP-Cy5.5; BioLegend, cat#344710), CD19 (CD19-APC; BioLegend, cat#302212), CD20 (CD20-PE; BioLegend, cat#302306), CD25 (CD25-APC; BioLegend, cat#302610), CD127 (CD127-PE; BioLegend, cat#351304), and PD-1 (PD-1-BV421; BioLegend, cat#329920). Cells stained by antibodies were resuspended in 1x PBS, and analyzed by flow cytometry using BD LSR Fortessa cell analyzer. Flow cytometry data were analyzed using FlowJo (version 10.4.0). Accordingly, B cells and Treg cells were selected as CD19^+^ CD20^+^ and CD3^+^ CD4 ^+^ CD25^+^ CD127^−^ subsets, independent CD3^+^ clustering of either CD4^+^ or CD8^+^ T cells with PD-1^+^ used for identifying PD-1 expression respectively from lymphocytes. Flow cytometry gating strategies for CD3+CD4+/CD3+CD8+ T cell populations see **Supplementary Figure 2**.

### Statistical analyses

Data were analyzed using SPSS (version 21.0 for Windows, Chicago, IL, USA). Continuous variables were shown as medians and interquartile ranges (IQR) unless stated otherwise. Mann-Whitney U test was used for the comparison among groups. The ROC curve was generated to analyze the predictive probability, and the area under the ROC curve (AUC) was calculated. All tests were two-tailed and *P* values of < 0.05 were considered to indicate a significant difference.

## Results

### Patient characteristics

Seventy-eighty out of nighty-five eligible patients with complete serum cytokines were included in this analysis. The demographic and clinical characteristics of patients were shown in **Table 1**. In all patients, 30 individuals (38.5%) achieved HBsAg seroclearance. The mean age in the cured group was 40.6 (36-45) years old, which was younger than that of the uncured group. There was a comparable gender distribution in both groups with which the majority were male 67 (85.9%): 26 (86.7%) in the cured group and 41 (85.4%) in the uncured group. Baseline HBsAg was significantly lower in the cured group (311.6 (39.0-485.3) than that in the uncured group (460.1 (185.2-741.3), P =0.031). No significant differences in the levels of white blood cell (WBC) (*P* = 0.077), neutrophil (*P* = 0.132), hemoglobin (*P* = 0.124), platelets (*P* = 0.204), albumin (*P* = 0.224), ALT (*P* = 0.305), AST (*P* = 0.486) and total bilirubin(TBIL)(*P* = 0.461) were found between two groups.

**Table 1:** Baseline demographic and clinical characteristics of both groups.

Characteristics	All patients (N =78)	Cured (N =30)	Uncured (N =48)	P*
Age, mean median (IQR), years	42.3 (37-48)	40.6 (36-45)	44.3 (38-49)	0.042
Gender				
Male, n (%)	67 (85.9)	26 (86.7)	41 (85.4)	0.592
HBsAg, median (IQR), log10 IU/ml	402.9 (129.1-702.1)	311.6 (39.0-485.3)	460.1 (185.2-741.3)	0.031
Laboratory tests				
White blood cell count, median (IQR), 10^9^/L	6.34 (5.53-7.11)	6.00 (5.10-6.67)	6.57 (5.81-7.37)	0.077
Neutrophil count median (IQR), %	3.57 (2.93-4.01)	3.36 (2.48-3.96)	3.71 (3.02-4.16)	0.132
Hemoglobin, median (IQR), g/L	155 (148-164)	151 (144-161)	157 (150-165)	0.124
Platelets, median (IQR), g/L	226 (185-262)	215 (169-254)	234 (197-263)	0.204
Albumin, median (IQR), g/dL	48.1 (46.8-49.8)	48.4 (47.5-49.7)	47.9 (46.3-49.8)	0.224
ALT, median (IQR), IU/L	26.9 (18.3-34.0)	28.0 (19.5-36.8)	26.2 (18.0-32.5)	0.305
AST, median (IQR), IU/L	23.9 (20.0-26.8)	23.6 (20.1-24.0)	24.1 (20.0-27.3)	0.486
Total bilirubin, median (IQR), μmol/L	14.3 (9.7-17.1)	15.2 (9.8-19.0)	13.7 (9.7-14.9)	0.461

*Comparisons were performed between these two groups using the Mann-Whitney U test.

### Kinetics of cytokine expression in response to the combination therapy with NAs and Peg-IFNα

**Figure 1:**
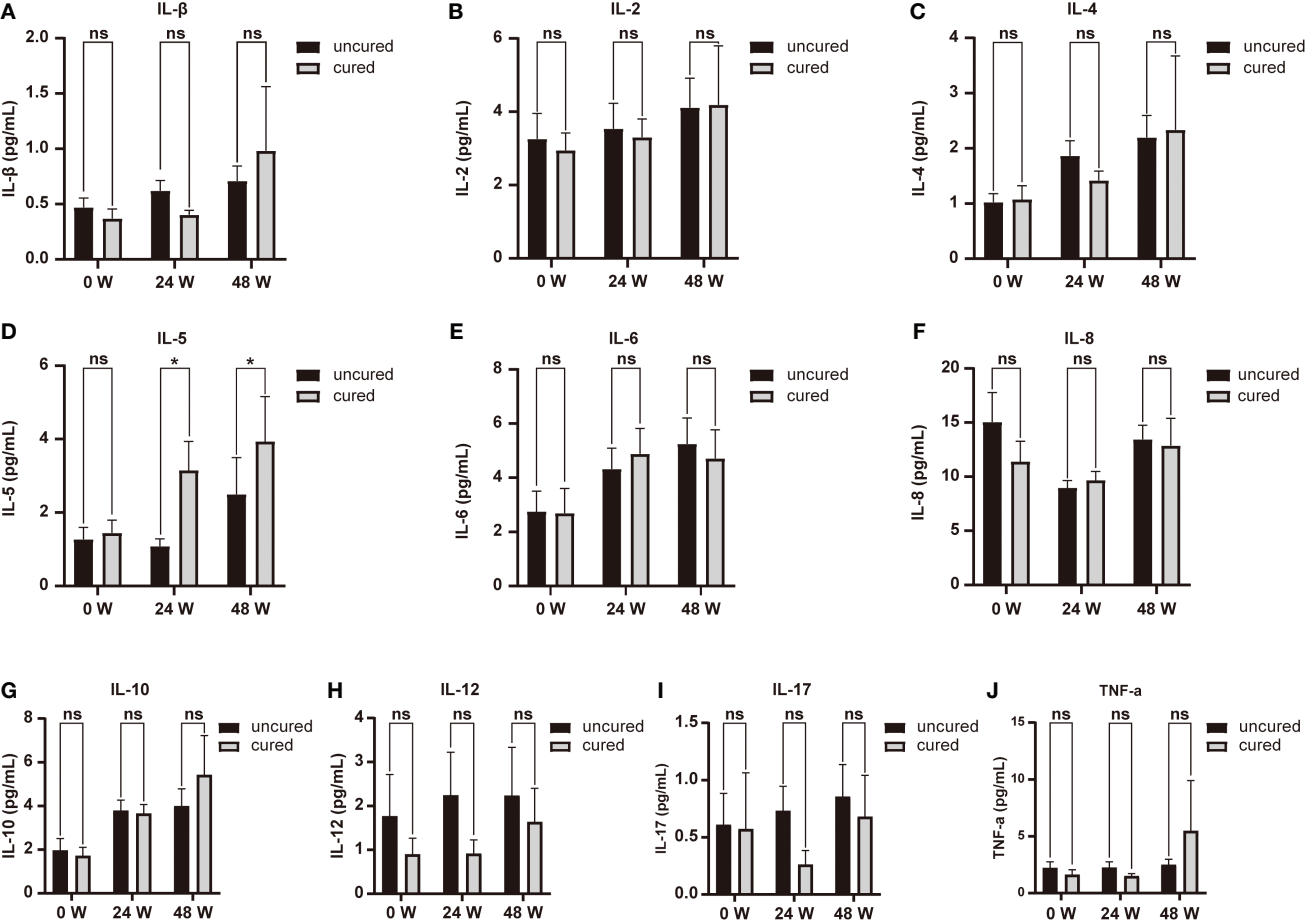
Serum cytokines expression during combination therapy with NAs and Peg-IFNα. The expressions of **(A)** IL-1β, **(B)** IL-2, **(C)** IL-4, **(D)** IL-5, **(E)** IL-6, **(F)** IL-8, **(G)** IL-10, **(H)** IL-12, **(I)** IL-17 and **(J)** TNF-α were analyzed at 0W, 24W and 48W in both groups. Data were presented as the mean ± SEM. ns, no significant; *, *P* < 0.05; 0W, the start of treatment; 24W, 24 weeks of treatment; 48W, 48 weeks of treatment.

The kinetics of cytokine expression in two groups were shown in **Figure 1**. The expressions of IL-1β, IL-2, IL-4, IL-10 and IL-12 showed similar overall upward trends throughout the course of treatment in all patients, with no significant difference (IL-1β: *P0W* = 0.307, *P24W* = 0.212, *P48W* = 0.082; IL-2: *P0W* = 0.979, *P24W* = 0.303, *P48W* = 0.124; IL-4: *P0W* = 0.938, *P24W* = 0.781, *P48W* = 0.118; IL-10: *P0W* = 0.845, *P24W* = 0.906, *P48W* = 0.579; IL-12: *P0W* = 0.508, *P24W* = 0.589, *P48W* = 0.459) (**Figures 1A, B, C, G, H**). The levels of IL-17 decreased at 24 weeks then increased at 48 weeks in the cured group while constantly elevated in the uncured group (IL-17: *P0W* = 0.497, *P24W* = 0.16, *P48W* = 0.126); (**Figure 1I**). IL-8 exhibited a downward trend before 24 weeks then increased at 48 weeks in both groups (IL-8: *P0W* = 0.704, *P24W* = 0.369, *P48W* = 0.479) (**Figure 1F**). The constantly elevated IL-6 expression was found in all patients before 24 weeks. Furthermore, IL-6 expression increased further until 48 weeks in the uncured group while it was decreased at 48 weeks in the cured group (IL-6: *P0W* = 0.244, *P24W* = 0.637, *P48W* = 0.246) (**Figure 1E**). In contrast to the persistently slow increase in the uncured group, TNF-α displayed a significant increase in the cured group from 24 weeks to 48 weeks (TNF-α: *P0W* = 0.618, *P24W* = 0.558, *P48W* = 0.059) (**Figure 1J**). IL-5 was notably increased in cured group during the whole course of treatment, while it decreased in the uncured patients at 24 weeks and then increased. Interestingly, the difference between the cured and the uncured groups was statistically significant (IL-5: *P0W* = 0.596, *P24W* = 0.033, *P48W* = 0.027) (**Figure 1D**).

### Correlation between IL-5 expression and HBsAg seroclearance

**Figure 2:**
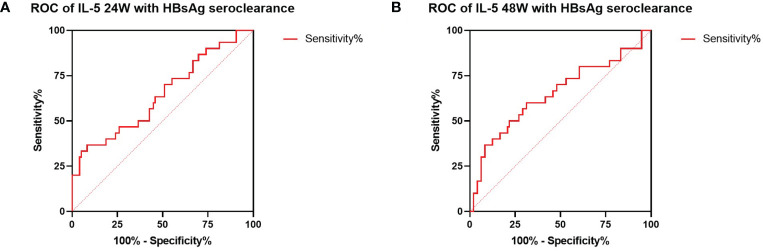
ROC analysis between IL-5 expression and HBsAg seroclearance. **(A)** ROC analysis showing the performance of IL-5 expression in distinguishing HBsAg seroclearance at 24W. AUC= 0.644 (95% CI, 0.515-0.773) (*P* = 0.034). **(B)** ROC analysis showing the performance of IL-5 expression in distinguishing HBsAg seroclearance at 48W. AUC=0.649 (95% CI, 0.517-0.781) (*P* =0.028). ROC, receiver operating characteristic; AUC, area under the curve; CI, confidence interval.

We also investigated the relationship between cytokines expression and HBsAg seroclearance. Serum levels of IL-5 were associated with HBsAg seroconversion between both groups at 24 and 48 weeks of treatment (**Figure 1D**). We thus performed ROC curve analysis. The results of the diagnostic accuracy of IL-5 expression to predict the rate for HBsAg seroconversion were shown in **Figure 2**. The AUCs presented by IL-5 expression at 24 and 48 weeks were 0.644 (95% CI, 0.515-0.773) with *P* < 0.05 and 0.649 (95% CI, 0.517-0.781) with *P* < 0.05 respectively.

### Immune cell subpopulations at different time points in response to the combination therapy of NAs and Peg-IFNα

**Figure 3:**
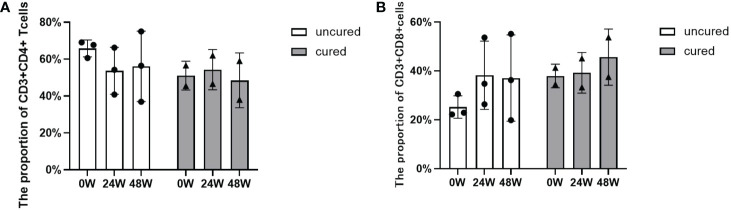
Dynamic changes of CD4^+^ T and CD8^+^ T cell subsets during combination therapy with NAs and Peg-IFNα. **(A)** The proportion of CD3^+^ CD4^+^ T cells at 0W, 24W and 48W. **(B)** The proportion of CD3^+^ CD8^+^ T cells at 0W, 24W and 48W. Data were presented as the mean ± SEM. 0W, the start of treatment; 24W, 24 weeks of treatment; 48W, 48 weeks of treatment.

As shown in **Figure 3A**, the proportion of CD4^+^ T cells were increased from the beginning of treatment to 24 weeks in the cured patients, then decreased to the basal level at 48 weeks, however, the uncured group showed an opposite trend, a decrease from 0 to 24 weeks after treatment, which was followed by a slow increase from 24 to 48 weeks. The similar patterns found in the CD8^+^ T cells were shown in **Figure 3B**. The proportion of CD8^+^ T cells changed less at 24 weeks after treatment in the cured group, and then increased from 24 to 48 weeks. Conversely, the uncured patients showed an elevation of CD8^+^ T cells within 24 weeks after therapy, which was decreased a little from 24 to 48 weeks.

**Figure 4:**
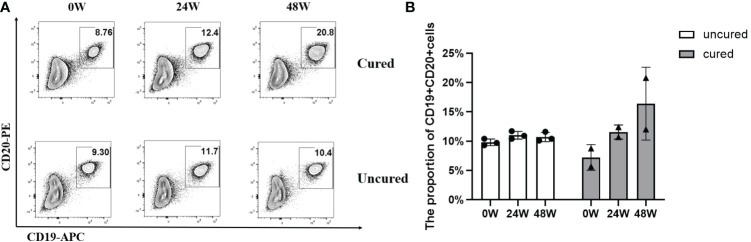
Frequency of B cells during combination therapy with NAs and Peg-IFNα. **(A)** Representative flow cytometric profiles showing the frequency of B cells (CD19^+^ CD20^+^) in PBMCs at different time points in both groups. APC, allophycocyanin; PE, phycoerythrin. **(B)** The proportion of CD19^+^ CD20^+^ T cells at 0W, 24W and 48W in both groups. Data were presented as the mean ± SEM. 0W, the start of treatment; 24W, 24 weeks of treatment; 48W, 48 weeks of treatment.

The effects of B cell subsets among CHB patients following combination treatment were also examined and results were shown in **Figure 4**. In both groups, B cell subsets showed similar increases until 24 weeks of treatment, then further increased in the cured group while decreased in the uncured group at 48 weeks.

**Figure 5:**
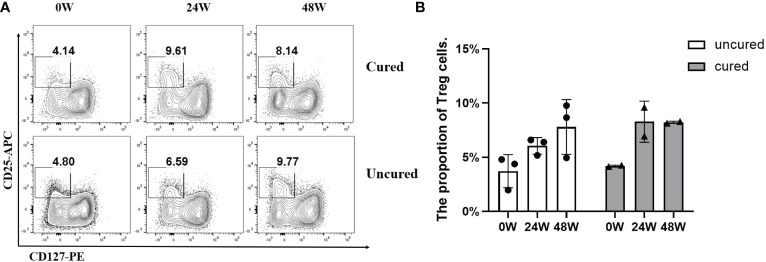
Frequency of Treg cells during combination therapy with NAs and Peg-IFNα. **(A)** Representative flow cytometric profiles showing the frequency of Treg cells in PBMCs at different time points in both groups. CD25^+^ CD127^-^ cells gated from CD3^+^ CD4^+^ cells were treated as Treg cells: CD3^+^ CD4^+^ CD25^+^ CD127^-^. APC, allophycocyanin; PE, phycoerythrin. **(B)** The proportion of Treg cells at 0W, 24W and 48W in both groups. Data were presented as the mean ± SEM. 0W, the start of treatment; 24W, 24 weeks of treatment; 48W, 48 weeks of treatment.

To explore the mechanisms underlying immunomodulation during combination treatment, we measured Treg population (**Figure 5**). The proportion of Treg cells in the uncured group was persistently elevated after treatment. In the cured group, this proportion was increased until 24 weeks then decreased at 48 weeks.

**Figure 6:**
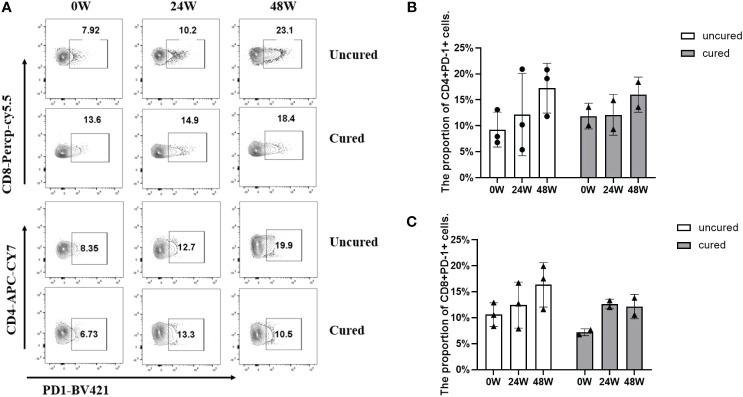
Frequency of CD4^+^ PD-1^+^ cells and CD8^+^ PD-1^+^ cells during combination therapy with NAs and Peg-IFNα. **(A)** Representative flow cytometric profiles showing the frequency of CD4^+^ PD-1^+^ cells and CD8^+^ PD-1^+^ cells in PBMCs at different time points in both groups. **(B)** The proportion of CD3^+^ CD4^+^ PD-1^+^ cells at 0W, 24W and 48W in both groups. **(C)** The proportion of CD3^+^ CD8^+^ PD-1^+^ cells at 0W, 24W and 48W in both groups. APC-Cy7, allophycocyanin-cyanine 7; PerCP-Cy5.5, Peridinin-Chlorophyll-Protein Complex- cyanine5.5; BV421, Brilliant Violet™421. Data were presented as the mean ± SEM. 0W, the start of treatment; 24W, 24 weeks of treatment; 48W, 48 weeks of treatment.

In the end, we examined PD-1 expressions in T cells from available patients (**Figure 6A**). As a result, the expressions reached to similar levels in both groups at 24 weeks, however, at 48 weeks, PD-1 expressions were higher from both CD4^+^ T cells (**Figure 6B**) and CD8^+^ T cells (**Figure 6C**) in the uncured group compared to that in the cured group, especially obvious from CD8^+^ subsets (**Figure 6C**).

## Discussion

In our recent study, we observed a high proportion of HBsAg seroclearance in NAs-experienced patients with HBV DNA negative treated with NAs and Peg-IFNα. Our study confirmed the efficacy of this combination therapy for reducing HBsAg level, especially in patients who received a ‘switch to’ combination therapy, which was consistent with the findings from previous studies (16, 17). NAs and Peg-IFNα combination therapy has both directly antiviral and indirectly immunomodulatory effects. Peg-IFNα can inhibit HBV transcription and reduce the production of viral antigens by enhancing the degradation of HBV pregenomic RNA and core particles or by modifying the epigenetic regulation of cccDNA. It can purge cccDNA from the nucleus by inducing deaminases and the nuclease ISG20 that modify and subsequently degrade cccDNA (18). Our study involved patients with inhibited HBV DNA and low levels of HBsAg after treatment with NAs. NAs therapy would first lead to strong suppression of viremia, thereby assisting restoration of HBV-specific CD8^+^ T cells (19), Peg-IFNα can then be more effective in enhancing antigen presentation to the immune system, activation of natural killer (NK) cells and other immune cells, increasing the production of cytokines (20). Here, we first performed a longitudinal analysis of cytokines subsets in NAs-experienced CHB patients with low HBsAg levels undergoing NAs and Peg-IFNα combination therapy.

In this study, we screened the levels of 10 cytokines in patients under NAs and Peg-IFNα combination therapy and analyzed their associations with HBsAg seroclearance. The present study did not show any differences among these cytokines between the cured and uncured patients throughout the course of treatment, except for IL-5. Serum IL-5 levels increased remarkably in cured group throughout the course of treatment and had a positive correlation with HBsAg seroclarance. IL-5 was originally defined as a T-cell-derived cytokine, mainly produced by activated Th2 cells, that triggers activated B-1 and B-2 cells for terminal differentiation into antibody-secreting plasma cells (21, 22). IL-5 receptor (IL-5R) comprises α and βc chains. IL-5 specifically binds to IL-5Rα and induces the recruitment of βc to IL-5Rα (23). B-1 cells constitutively express the IL-5Rα and give rise to Ab-producing cells in response to IL-5 stimulation (24). The higher expression of IL-5 found in our patients is in line with the elevated trend of B cells in cured group.

It was reported that HBsAg level at 24 weeks can be used to predict HBsAg loss during Peg-IFNα therapy (25). The present study also showed the different change timepoint at 24 weeks between the cured and uncured groups. Treatment with Peg-IFNα led to increased expression levels of serum cytokine and immune cell subpopulations before 24 weeks. Our study involved patients with inhibited HBV DNA and low levels of HBsAg after treatment with NAs. Their immune functions were restored after NAs treatment, such as those of CD8^+^ T cells. Peg-IFNα can then be added to accelerate the decline of circulating and intrahepatic viral antigens and allow activating immune cells and inducing increased production of cytokines (20). Moreover, protracted antigenic stimulation may result in B-cell activation and differentiation to immunoglobulin-producing cells (IPC) (26), potentially leading to a rapid decrease of HBsAg before 24 weeks of treatment.

On the other hand, due to the hyperresponsiveness of immunosuppressive cells (27), the cellular immune response in chronic HBV infection is exhausted (28). Regulatory T cells (Tregs) act as primary immunosuppressive cells in chronic HBV infection and play a critical role in coordinating immunologic tolerance to both self and foreign antigens by inhibiting HBV-specific responses of CD4^+^ T cells and CD8^+^ T cells. Depletion of Tregs *in vivo* has been shown to enhance the functionality of HBV-specific CD8+ T cells and accelerate the clearance of HBsAg (29). In the current study, Tregs were also activated before 24 weeks, whereas its expression was downregulated in the cured patients and continued unregulated in the uncured patients after 24 weeks. In terms of the PD-1 CD4^+^ T cells and PD-1 CD8^+^ T cells observed in our study, the uncured patients had higher frequencies of PD-1^+^CD4^+^ T cells after 24 weeks of treatment. Higher expression of PD-1 has been demonstrated to confer the exhaustion of HBV-specific CD4^+^ T cells and CD8^+^ T cells (30, 31). Blocking the interactions of PD-1 and its ligand PD-L1 *in vitro* has been shown to reverse the exhaustion of viral-specific T cells (32), restoring the functionality of viral-specific B cells (33) and Tregs (34). These findings are similar to the results of the current study. Therefore, we speculate that the cured patients demonstrate a better immune response to Peg-IFNα, especially the immunity activity of CD4^+^ T cell and B cells, inducing a more rapid increase of serum IL-5 and a decline of HBsAg. Meanwhile, the continued higher expression of Tregs in the uncured patients after 24 weeks of treatment may weaken the immune function of CD8^+^ T cells and B cells and therefore lead to a higher expression of HBsAg in some uncured patients.

To our knowledge, this is the first study to demonstrate the association between IL-5 and HBsAg seroclearance and provide new insights into the immune mechanisms of “functional cure” in NAs-experienced patients treated with NAs combined with Peg-IFNα. The regulated immune mechanisms involved in HBsAg seroclearance are complex. Due to the limited availability of PBMC samples, in addition to the dynamic change of B cells, CD4^+^ T cells, CD8+ T cells, and Treg cells during the course of treatment observed in our study, other immune cell subpopulations, like DC, NK, and T Helper cell and B cell subsets, would be helpful to explore the immune mechanism. Future longitudinal studies are hence needed to address this issue.

In conclusion, we found that serum IL-5 levels were closely associated with HBsAg seroclearance in NAs-experienced CHB patients treated with NAs combined with Peg-IFNα. Serum IL-5 levels after 24 weeks of treatment could predict HBsAg seroclearance and response to combination therapy with NAs and Peg-IFNα.

## Data availability statement

The original contributions presented in the study are included in the article/**Supplementary Material**. Further inquiries can be directed to the corresponding authors.

## Ethics statement

The studies involving human participants were reviewed and approved by the Research Ethics Committee of the Third Affiliated Hospital of Sun Yat-sen University. The patients/participants provided their written informed consent to participate in this study.

## Author contributions

QZ conceived of this study. QZ and PW performed the statistical analyses and wrote the manuscript. QZ, QT, and ZG supervised the writing process and revised the manuscript. PW and ZM contributed to the collection of data. All authors contributed to the article and approved the submitted version.
