# Supplementary figures and images for: Serum IL-5 levels predict HBsAg seroclearance in patients treated with Nucleos(t)ide analogues combined with pegylated interferon
Source: Front Immunol. 2023 Jan 5;13:1104329. doi: 10.3389/fimmu.2022.1104329 (PMC9849374; doi:10.3389/fimmu.2022.1104329)

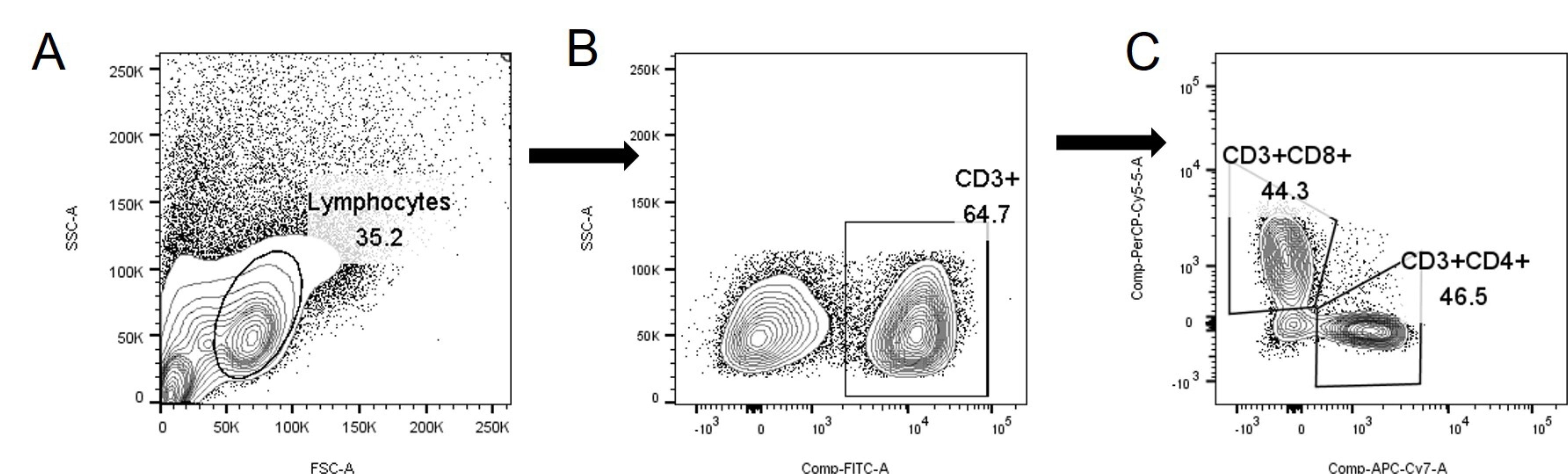

Supplement: Supplementary file 1 [file Image_1.tif]

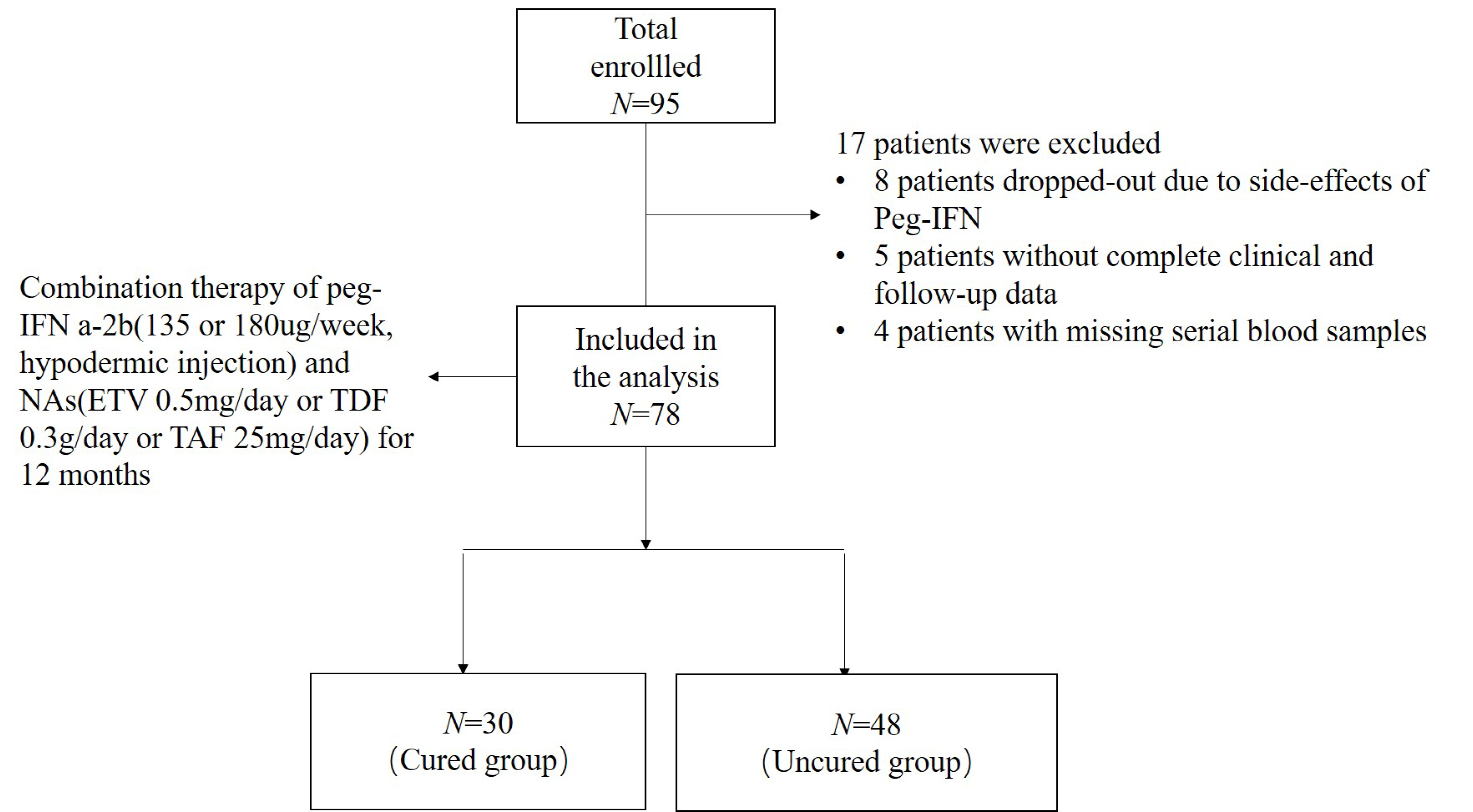

Supplement: Supplementary file 2 [file Image_2.tif]
